# Integrated bioinformatic analysis reveals *NOS2* as a novel ferroptosis-related biomarker for pre-eclampsia

**DOI:** 10.1186/s12884-023-06051-0

**Published:** 2023-10-10

**Authors:** Shuangming Cai, Shan Huang, Wenni Zhang, Huanshun Xiao, Danfeng Yu, Xuan Zhong, Pei Tao, Yiping Luo

**Affiliations:** Medical Intensive Care Unit, Guangdong Women and Children Hospital, Guangzhou, Guangdong China

**Keywords:** Pre-eclampsia, Ferroptosis, WGCNA, Differential gene expression, Biomarker, NOS2, GEO

## Abstract

**Background:**

Pre-eclampsia (PE) is a common condition in pregnancy; however, methods for early diagnosis and effective treatment options are lacking. Ferroptosis is a newly identified iron-dependent cell death pathway. The aim of this study was to investigate the role of ferroptosis-related genes in PE, the underlying mechanism, and their potential diagnostic value using a bioinformatics approach.

**Methods:**

We downloaded the GSE48424 and GSE98224 datasets from the Gene Expression Omnibus database. Differentially expressed genes (DEGs) between PE and healthy pregnancy samples were identified in the GSE48424 dataset and subjected to weighted gene co-expression network analysis; the most relevant modules were intersected with known ferroptosis-related genes to distinctly identify the role of ferroptosis in PE. We further searched transcription factors and microRNAs that are predicted to regulate these ferroptosis-related genes, and patients in the GSE48424 dataset were divided into two groups according to high or low expression of the key ferroptosis-related genes associated with PE. To obtain robust key ferroptosis-related genes in PE, we validated their expression levels in the external dataset GSE98224. Finally, the reverse transcription-quantitative polymerase chain reaction (RT-qPCR) assay was utilized to access the expression of these genes in the PE and normal blood samples.

**Results:**

Six ferroptosis-related genes involved in PE were obtained by overlapping 3661 genes most associated with PE, 565 DEGs between PE and normal samples, and 259 known ferroptosis-related genes. Among these genes, patients with PE displaying lower expression levels of *NOS2* and higher expression levels of *PTGS2* had a higher ferroptosis potential index. The expression pattern of *NOS2* was consistent in the GSE48424 and GSE98224 datasets. RT-qPCR data confirmed that *NOS2* expression was more significantly elevated in patients with PE than in those with a normal pregnancy.

**Conclusions:**

Our study explored the diagnostic value of ferroptosis-related genes in PE, and identified *NOS2* as the key gene linking ferroptosis and PE, suggesting a new candidate biomarker for early PE diagnosis.

## Background

Pre-eclampsia (PE), characterized by organ damage and hypertension after 20 gestational weeks, has a prevalence of 2–8%. PE is associated with short-term complications such as cardiac, hepatic, pulmonary, renal, and neurological dysfunction, and high maternal and neonatal mortality. Long-term complications include a higher risk of cardiovascular events [1, 2]. The abnormal migration and invasion of the extravillous trophoblast toward the uterine spiral arteries, which is the main cause of PE, lead to neovascularization and uteroplacental vascular resistance [3, 4]. However, the exact pathophysiology of PE is unknown, and early diagnosis and treatment are lacking. Early diagnosis facilitates rapid clinical intervention and minimizes maternal and fetal damage. Therefore, the pathogenesis of PE requires further analysis, and it is important to find reliable diagnostic markers.

Programmed cell death typically occurs in the biological development process. Although previous studies in this regard have mainly focused on autophagy and apoptosis, the role of ferroptosis has become a research hotspot in recent years. As another form of programmed cell death, ferroptosis is quite distinct from the classical programmed cell death forms (e.g., apoptosis) [5, 6], with iron-dependent lipid-peroxidative damage as the main mechanism. Ferroptosis has been shown to play a role in several diseases such as asthma, brain damage, cancer, ischemic heart disease, and acute kidney injury [7–11]. At present, there is mounting evidence suggesting that ferroptosis has been related to PE [12]. The pathophysiological processes of PE, including placental hypoxia-reperfusion, abnormal placental perfusion, a rich iron content in the trophoblast, and reduced glutathione peroxidation capacity, are all linked to conditions associated with ferroptosis [13, 14]. Some studies have recorded the lipid peroxidation of trophoblasts in placental injury [15]. The concentration of plasma iron in PE pregnancy is higher than that in normal pregnancy [16]. There are also several articles exploring the relationship between ferroptosis and PE using bioinformatics methods [17, 18]. We were wondering if we could find biomarkers that were diagnostic for PE using different bioinformatics methods.

Investigation of the relationship between ferroptosis-related genes and PE can enrich our understanding of PE etiology and guide the early detection of PE. Thus, the aim of this study was to identify diagnostic ferroptosis-related biomarkers for the early diagnosis of PE using bioinformatics analysis and validation assays with clinical samples .

## Methods

### Data source

We recruited 16 pairs of patients with PE matched with women undergoing a normal pregnancy from the Department of Obstetrics of Guangdong Women and Children Hospital (China). The Ethics Committee of Guangdong Women and Children’s Hospital approved the study (No. 202,101,012), and all participants provided informed consent. Inclusion and exclusion criteria are as follows: PE was defined as new onset of systolic blood pressure elevation of at least 140 mmHg, or diastolic blood pressure of at least 90 mmHg on at least two occasions that were at least 4 h apart. This would need to be accompanied by one or more of the following features: proteinuria (≥ 300 mg/24 h), maternal organ dysfunction (including renal, hepatic and neurological), hematological involvement, and uteroplacental dysfunction. Pregnant women with cardiovascular diseases, liver or kidney diseases, endocrine diseases, or fetuses with malformations or chromosomal abnormalities were excluded. The clinical features of the patients are shown in Table 1.

**Table 1:** Clinical characteristics of the patients

Variables	PE (n = 16)	Normal (n = 16)	*p*
Maternal age (yr)	31.5 ± 5.51	31.37 ± 3.05	0.937
Maternal weight (kg)	70.58 ± 13.73	64.24 ± 6.54	0.104
Maternal height (cm)	159 ± 5.41	158.38 ± 5.19	0.749
BMI (kg/m^2^)	27.76 ± 4.01	25.49 ± 3.23	0.098
Parity, n			
Nulliparous	7	5	0.379
Multiparous	9	11	
Gravidity, n			
1–2	12	10	0.446
>2	4	6	
Gestational age at delivery (days)	247 ± 24.45	275.69 ± 6.25	0.000
systolic blood pressure (mm Hg)	168 ± 9.00	117.13 ± 7.14	0.000
diastolic blood pressure (mm Hg)	107.19 ± 11.01	70.88 ± 3.95	0.000
Urine dipstick, n			
Negative	0	16	0.01
1+	4	0	
2+	11	0	
3+	1	0	
4+	0	0	
24 h uPro (mg)	6188.47 ± 4207.13	N/A	
Birth weight (g)	2470.63 ± 822.36	3196.88 ± 263.00	0.002
BMI = body mass index, N/A = not applicable.			

Transcriptome data from patients with PE and normal pregnancies were downloaded from the GSE48424 dataset, including 18 control and 18 PE blood samples, and the GSE98224 dataset, including 18 control and 30 PE placenta tissue samples, of the Gene Expression Omnibus (GEO) (http://www.ncbi.nlm.nih.gov/geo) database to screen for ferroptosis-related genes involved in PE. Ferroptosis scores of the control and PE groups in the GSE48424 dataset were calculated by single-sample gene set enrichment analysis (ssGSEA) using a ferroptosis-related gene set from the FerrDb database (www.zhounan.org/ferrdb) [19].

### Differentially expressed gene (DEG) identification and functional analysis

We used the “limma” package in R (version 4.0.0) to identify the DEGs between PE and control samples with *p*-value < 0.05 and |fold change (FC)| >1.5. DEG function, interactions among enriched biological processes, and pathways were analyzed using Metascape [20]. We constructed a sample clustering tree map from the GSE48424 dataset.

### Weighted gene co-expression network analysis (WGCNA) and identification of the most relevant module associated with PE

To eliminate outliers and ensure the best soft threshold depending on gene expression profiles and patient grouping (control and PE groups), we performed WGCNA using R software package. We used Pearson correlation coefficients to determine the most relevant module associated with PE.

### Identification of potential diagnostic ferroptosis-related biomarkers in PE

DEGs, ferroptosis-related genes downloaded from the FerrDb database, and genes in the most relevant module identified by WGCNA were intersected to identify candidate ferroptosis-related genes involved in PE. Subsequently, the diagnostic value of candidate ferroptosis-related genes involved in PE was assessed by construction of receiver operating characteristic (ROC) curves. Candidate ferroptosis-related genes with area under the ROC curve (AUC) values greater than 0.7 were identified as potential diagnostic ferroptosis-related biomarkers in PE.

The Harmonizome database was used to search for transcription factors (TFs) that regulate the expression of the candidate diagnostic ferroptosis-related biomarkers in PE. The regulatory network of microRNAs (miRNAs) and TF diagnostic biomarkers was visualized using Cytoscape. Potential diagnostic ferroptosis-related biomarkers in PE were captured into the miRWalk database to screen for miRNAs targeting the diagnostic biomarkers (score > 0.9).

Patients with PE in the GSE48424 dataset were separated into high- and low-expression groups according to the median expression value of each diagnostic biomarker. The ferroptosis potential index (FPI) was calculated based on the expression levels of genes in the core ferroptosis machinery by ssGSEA, including the positive regulators *NFE2L2, LPCAT3, GPX4, NCOA4, ACSL4, SLC3A2, ALOX15, SLC7A11, NOX3, NOX1, NOX5*, and *NOX4*, and the negative regulators *COQ10B, HMGCR, FDFT1*, and *COQ10A*, as described in a previous study [21]. Thereafter, candidate key ferroptosis-related genes were identified according to a significant difference between the low- and high-expression groups. Gene expression was validated in the external dataset GSE98224 to obtain robust key ferroptosis-related genes in PE. The biological functions of key ferroptosis-related genes in PE were analyzed by GSEA.

### Western blot analysis

Placental tissue samples (1 × 1 × 1 cm3) were randomly collected from the basal plate (2 cm from the periphery, avoiding placental infarcts) immediately after delivery, and washed in cold normal saline to remove any contaminating blood. The tissue was immediately frozen at − 80 °C for immunoblotting experiments.

40 mg of tissue from different groups were lysed with RIPA buffer. Protein concentrations were quantified by BCA kit. Total protein (25 µg) was separated by SDS-PAGE, and then transferred to PVDF membranes which were clipped to the appropriate size covering all the sample lanes prior to transfer. The PVDF membranes were blocked with 5% skim milk and incubated with anti-NOS2 antibody (1:1000 dilution; Proteintech Group; No: 80517-1-RR) and anti-β-actin antibody (1:5000 dilution; Abcam; No: ab8226) overnight at 4 °C. The membranes were eluted three times with TBST for 10 min each, followed by incubation with 1:5000 HRP-conjugated secondary antibodies at room temperature for 1 h. The immunoreactive bands were visualized with chemiluminescence detection reagents by Bio-RAD camera system and analyzed by ImageJ software for relative expression of proteins.

### Validation with reverse transcription-quantitative polymerase chain reaction (RT-qPCR)

All patient samples were collected at the Department of Obstetrics, Guangdong Women and Children Hospital (Guangzhou, China). Venipunctures were performed, and 3 milliliters (ml) of whole blood was collected into 5-ml EDTA-coated Vacutainer tubes before any medical interventions. All samples were stored in a freezer at − 80 °C for future study.

TRIzol reagent (Tiangen, Beijing, China) was used to extract the total RNA from patient samples. The Revert Aid RT-PCR system was then used to perform reverse transcription to synthesize cDNA, and qPCR was performed on the ABI 7500 Real-Time PCR System (Roche, Penzberg, Germany) by mixing cDNA, primers, and the Rox Reference Dye. The conditions for qPCR were as follows: 40 cycles of denaturation (95 °C, 10 s), annealing (55 °C, 20 s), and extension (72 °C, 35 s). The primer sequences were shown in Table 2. The mRNA expression levels were calculated using the ΔΔCT method with *GAPDH* as a reference.

**Table 2:** Primer sequences for RT-qPCR.

Gene	Primer sequences (5′-3′)
*PTGS2*	CTCAGCCATACAGCAAATCCT (forward)
	CCGGGTACAATCGCACTTAT (reverse)
*GCH1*	CCTGGAAGCTGTTGCCTTAT (forward)
	TGTGTTTCTGTGGAGGAGTTG (reverse)
*HIF1A*	CCAGTTACGTTCCTTCGATCAG (forward)
	GTAGTGGTGGCATTAGCAGTAG (reverse)
*BACH1*	CTCCGCAGGTATCAAGGAAAT (forward)
	TAAAGAAGGCAAGGCCAGAG (reverse)
*NOS2*	CTCAGCCTCATTCCTGCTTTA (forward)
	GACCTGTGCCTTGAGAACTT (reverse)
*GAPDH*	CAAGAGCACAAGAGGAAGAGAG (forward)
	CTACATGGCAACTGTGAGGAG (reverse)

### Statistical analysis

All data were analyzed using R software version 4.0.0. Boxplots and volcano plots were plotted by ggplot2 package in R (version: 3.3.2). The significance of differences between PE and normal groups was calculated by ggsignif package (version 0.6.0). T-test was used to compare ferroptosis scores between PE and normal samples. A *p*-value < 0.05 was considered to indicate statistical significance.

## Results

### DEGs in PE are associated with immune and lipid metabolism

**Fig. 1:**
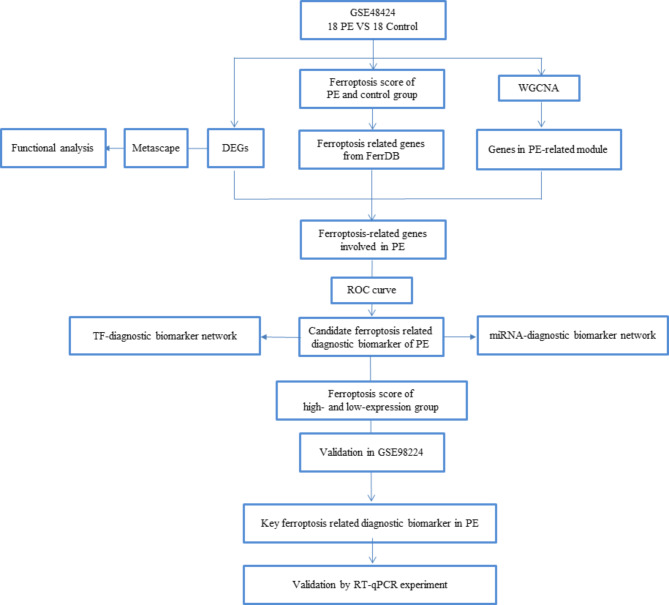
Study workflow. PE, pre-eclampsia; WGCNA, weighted gene co-expression network analysis; DEG, differentially expressed gene; ROC, receiver operating characteristic; TF, transcription factor; miRNA, microRNA.

**Fig. 2:**
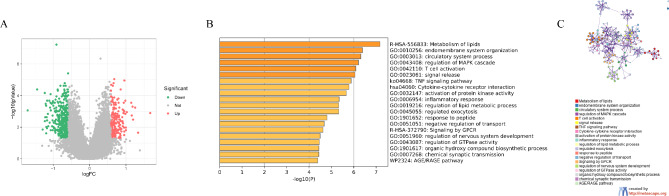
Identification and functional analysis of differentially expressed genes (DEGs) between pre-eclampsia (PE) and control samples. (**A**) Volcano plot of DEGs between PE and control samples; DEGs were screened according to a threshold of *p*-value < 0.05 and |fold change|> 1.5. (**B**) Bar chart showing the top 20 enriched terms determined by Metascape. (**C**) Interactive network of the top 20 enrichment terms

The workflow of the current study is displayed in Fig. 1. We identified 565 DEGs, including 378 downregulated and 187 upregulated genes in PE compared with control samples (Fig. 2A). Metascape confirmed that these DEGs were largely enriched in immune- and lipid metabolism-related biological processes, including the metabolism of lipids, regulation of the mitogen-activated protein kinase cascade, T cell activation, inflammatory response, cytokine–cytokine receptor interaction, tumor necrosis factor signaling pathway, and regulation of lipid metabolic process (Fig. 2B). Metascape further revealed that these biological processes and pathways interact (Fig. 2C), indicating that the pathogenesis of PE is complex and regulated by cross-talk among multiple biological processes and pathways.

### Most relevant module associated with PE identified by WGCNA

**Fig. 3:**
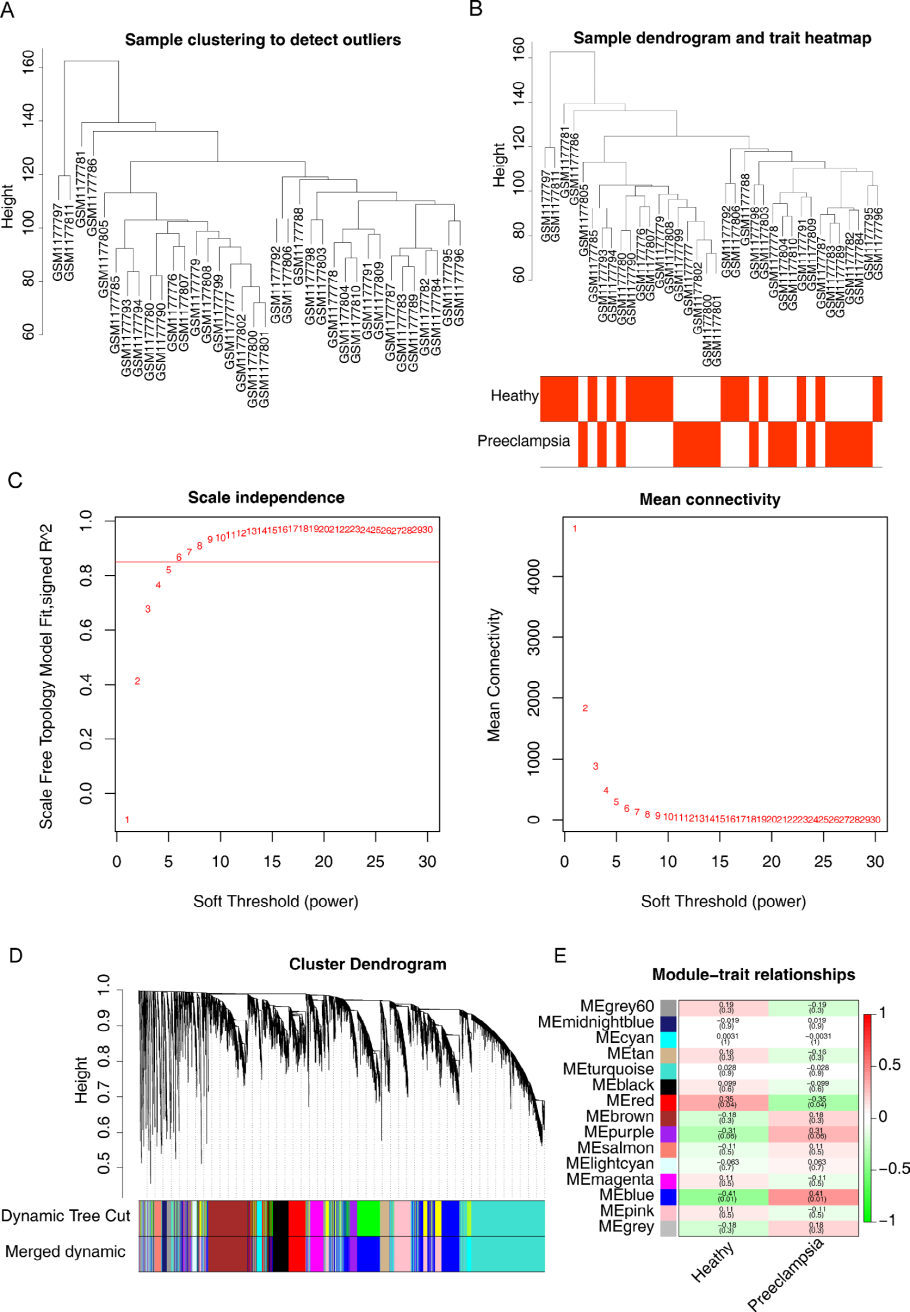
Screening the key gene modules correlated with pre-eclampsia (PE) by weighted gene co-expression network analysis (WGCNA). (**A** and **B**) Sample clustering to detect outliers. There were no samples outside of clusters. (**C**) Scale-free index calculated under different soft thresholds. The average connectivity is calculated at different soft thresholds. (**D**) Gene clustering tree (tree view) obtained from the hierarchical clustering of adjacency correlation. The colored rows below the tree represent the gene modules identified by the dynamic cutting tree method. (**E**) Heatmap of the correlation between module eigengenes and traits (healthy pregnancy and PE). Each row corresponds to a module. Each column corresponds to a trait. Each cell contains the corresponding correlation coefficient and *p* value

No outlier sample was detected in the GSE48424 dataset (Fig. 3A). The trait heatmap and sample dendrogram are shown in Fig. 3B. The optimal soft threshold power was identified as 6, for which the R^2^ was 0.85 (Fig. 3C). After merging similar modules, we identified 15 modules from the co-expression network (Fig. 3D). The ME (blue) module, comprising 3661 genes, was the most relevant module associated with PE (correlation coefficient = 0.41, *p* = 0.01) and was thus selected for further analysis.

### Identification of five diagnostic ferroptosis-related biomarkers in PE

**Fig. 4:**
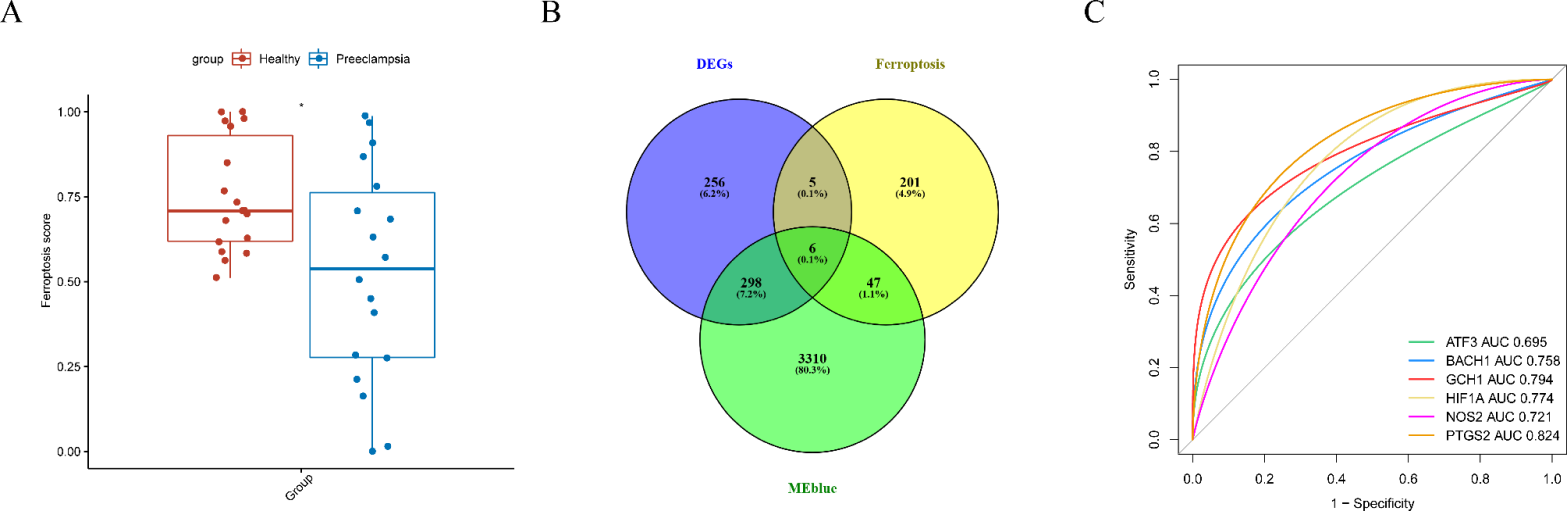
Identification of diagnostic ferroptosis-related biomarkers in pre-eclampsia (PE). (**A**) Ferroptosis score calculated by single-sample gene set enrichment analysis between PE and control samples. (**B**) Venn diagram showing six overlapping candidate ferroptosis-related genes and differentially expressed genes (DEGs) involved in PE. (**C**) Receiver operating characteristic curve analysis to evaluate the diagnostic value of the six candidate ferroptosis-related genes in PE.

We compared the ferroptosis scores between PE and control samples by ssGSEA. The PE group had significantly (*p* < 0.05) lower ferroptosis scores than the control group (Fig. 4A), indicating that ferroptosis is involved in the etiology of PE. Six candidate ferroptosis-related genes involved in PE were identified by overlapping the 565 DEGs, 3661 genes identified in the most relevant WGCNA module, and 259 known ferroptosis-related genes (Fig. 4B). ROC curve analysis was applied to evaluate the diagnostic value of the six candidate ferroptosis-related genes. The AUC values for *PTGS2, GCH1, HIF1A, BACH1*, *NOS2*, and *ATF3* were 0.824, 0.794, 0.774, 0.758, 0.721, and 0.695, respectively (Fig. 4C). Therefore, *PTGS2, GCH1, HIF1A, BACH1*, and *NOS2* were identified as candidate diagnostic ferroptosis-related biomarkers in PE.

### Regulatory network of diagnostic ferroptosis-related biomarkers in PE

**Fig. 5:**
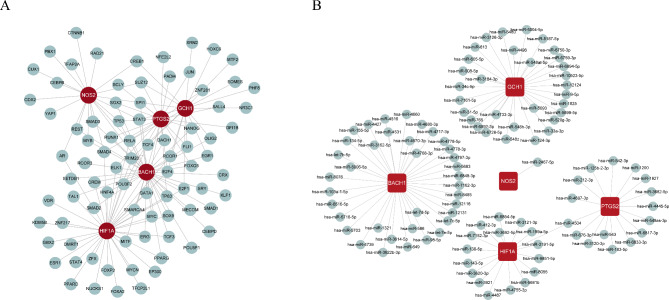
Regulatory network of potential diagnostic ferroptosis-related biomarkers in pre-eclampsia. (**A**) Regulatory network of transcription factor–diagnostic biomarkers constructed and visualized by Cytoscape. (**B**) Regulatory network of microRNA–diagnostic biomarkers visualized by Cytoscape

The ChEA Transcription Factor Targets function in Harmonizome was used to screen TFs regulating the expression of *PTGS2, GCH1, HIF1A, BACH1*, and *NOS2*. A regulatory network of *PTGS2, GCH1, HIF1A, BACH1, NOS2*, and 85 TFs was constructed and visualized using Cytoscape (Fig. 5A). *PTGS2, GCH1, HIF1A, BACH1*, and *NOS2* expression was found to be regulated by both specific and common TFs. We also detected the miRNAs targeting these five biomarkers using the miRWalk database and constructed the miRNA–mRNA network using Cytoscape. We found 31 miRNA–*GCH1* pairs, 37 miRNA–*BACH1* pairs, a has-miR-2467-5p–*NOS2* pair, 17 miRNA–*HIF1A* pairs, and 16 miRNA–*PTGS2* pairs (Fig. 5B).

### Identification of *NOS2* as the key ferroptosis-related gene in PE

**Fig. 6:**
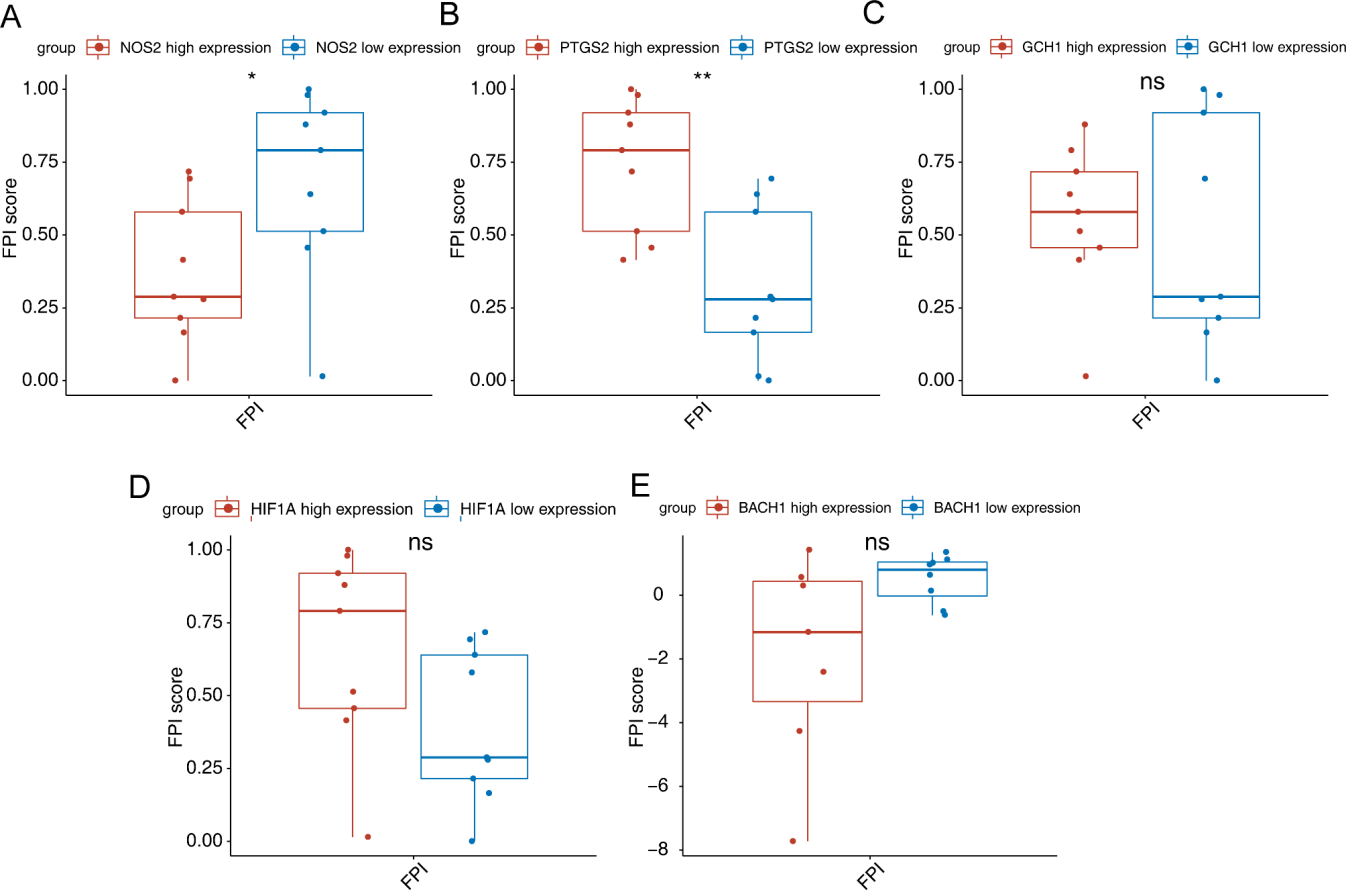
Ferroptosis potential index (FPI) of high- and low-expression groups calculated by single-sample gene set enrichment analysis (ssGSEA). (**A**) Box plot showing the FPI score between high- and low-*NOS2* expression groups. (**B**) Box plot showing the FPI score between high- and low-*PTGS2* expression groups. (**C**) Box plot showing the FPI score between high- and low-*GCH1* expression groups. (**D**) Box plot showing the FPI score between high- and low-*HIF1A* expression groups. (**E**) Box plot showing the FPI score between high- and low-*BACH1* expression groups. ns, not significant; *p < 0.05; **p < 0.01

**Fig. 7:**
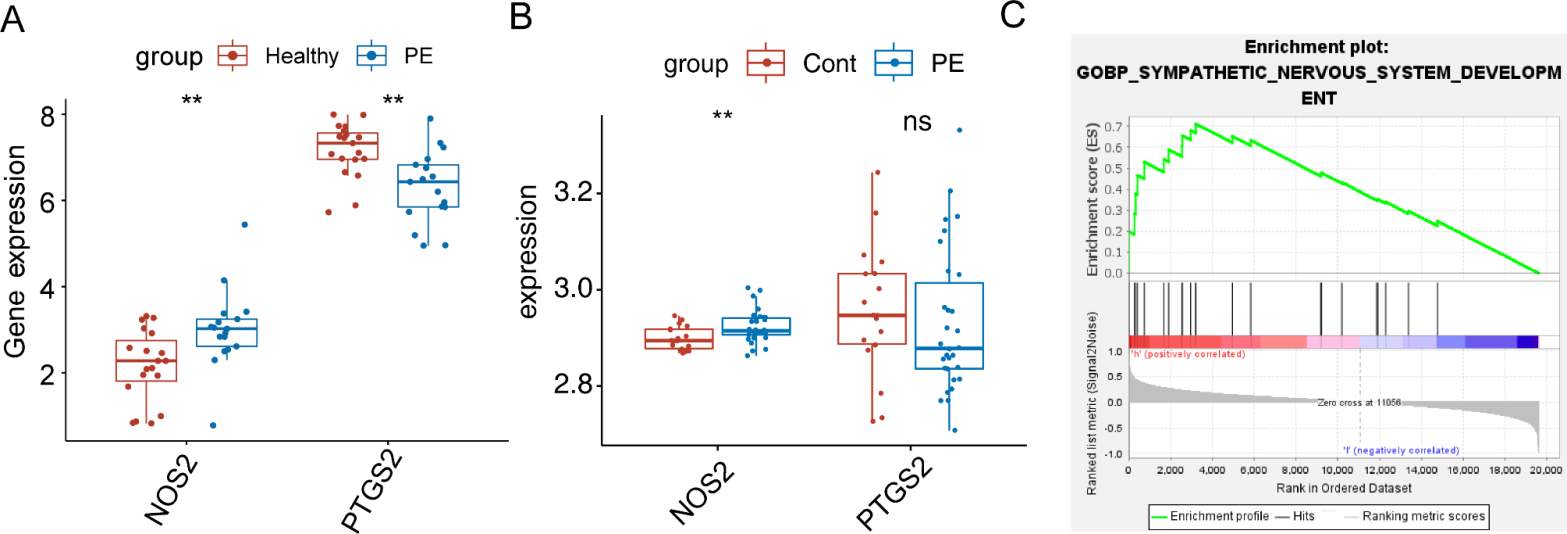
Gene expression and gene set enrichment analysis of ferroptosis-related genes. (**A**) Box plot showing gene expression of *NOS2* and *PTGS2* between pre-eclampsia (PE) and healthy groups in the GSE48424 dataset. (**B**) Box plot showing gene expression of *NOS2* and *PTGS2* between PE and control groups in the GSE98224 dataset. (**C**) Potential mechanism of the involvement of *NOS2* in PE assessed by gene set enrichment analysis

To investigate the importance of these six candidate biomarkers in mediating the ferroptosis-related etiology of PE, we divided pregnant women with PE into low- and high-expression groups according to the median expression level of each biomarker. The low-*NOS2* group had a significantly (*p* < 0.05) higher FPI than that of the high-*NOS2* group (Fig. 6A),and the high-*PTGS2* group had a significantly (*p* < 0.05) higher FPI than the low-*PTSG2* group(Fig. 6B). No significant difference was detected between low- and high-expression groups with respect to the levels of *GCH1, HIF1A*, and *BACH1* (Fig. 6C–E), indicating that *NOS2* and *PTGS2* have greater contributions to the ferroptosis-related etiology of PE. Furthermore, the abundance of *NOS2* was higher in the PE group, whereas the abundance of *PTGS2* was higher in the control group in the GSE48424 dataset (Fig. 7A). The expression pattern of *NOS2* was validated in the GSE98224 dataset (Fig. 7B). Thus, *NOS2* was considered to be a key ferroptosis-related gene in PE. ssGSEA showed that sympathetic nervous system development was significantly enriched in the high-*NOS2* expression group (*p* < 0.01, Fig. 7C), suggesting the potential mechanism by which *NOS2* is involved in PE.

**Fig. 8:**
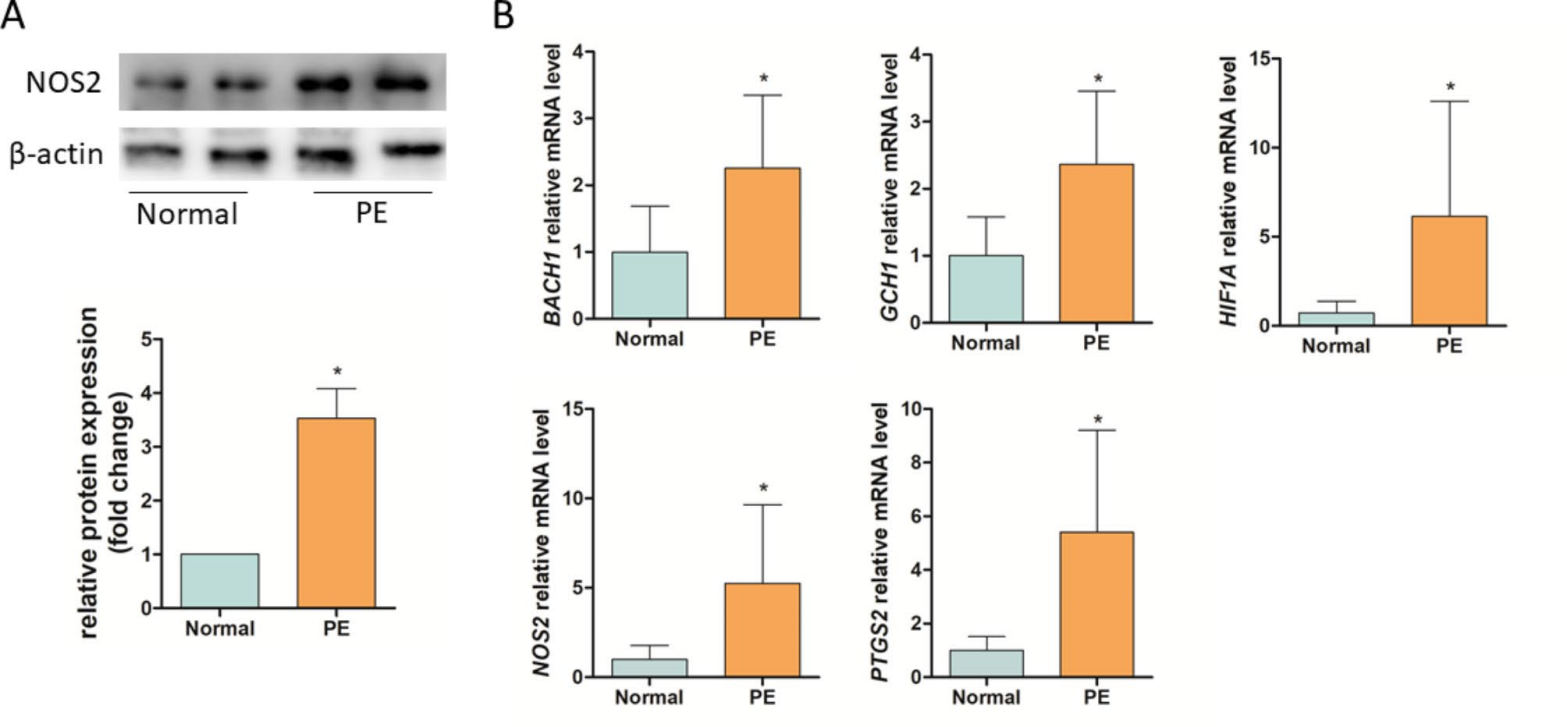
Protein expression of *NOS2* in placental tissue and relative mRNA expression levels of the identified potential diagnostic ferroptosis-related genes in blood from women undergoing a normal pregnancy (control) and pre-eclampsia (PE) patients. (**A**) Western blot of *NOS2* and β-actin in placental tissue and relative protein expression (n = 10 per group). (**B**) Relative mRNA expression levels of the identified potential diagnostic ferroptosis-related genes in blood samples using RT-qPCR analysis (n = 16 per group). *p < 0.05 compared with the normal group

We evaluated the protein level of *NOS2* in placental tissue using Western blot, and the results showed *NOS2* expression was significantly elevated in the PE group compared with the normal pregnancy group (Fig. 8A). Validation with RT-qPCR confirmed that the *NOS2* mRNA expression level was significantly elevated in patients with PE compared with that of pregnant women undergoing a normal pregnancy (Fig. 8B).

## Discussion

PE has dire consequences for both maternal and neonatal health. However, early diagnosis and radical treatment for PE are lacking as its etiology is not well-understood. As a novel regulated form of cell death, ferroptosis has recently been shown to play a role in the development of multiple diseases [22], making it a promising biomarker to aid in early diagnosis. This study investigated the diagnostic value of ferroptosis-related genes in PE.

We identified 565 DEGs that mainly participate in the lipid metabolism and immune processes. Previous studies reported that a large amount of energy required for placental and fetal development is provided by free fatty acids (FFAs). Lorentzen et al. [23] reported that the serum levels of FFAs such as linoleic acid, palmitic acid, and oleic acid are elevated in patients with PE, and lower levels of long-chain polyunsaturated fatty acids in either the placenta or maternal circulation have also been related to PE pregnancies [24–26]. Moreover, altered fatty acid oxidation may contribute to the pathophysiology of PE [27]. Accumulating evidence indicates that improper activation of the immune system may lead to the development of PE [28]. These findings suggested that lipid metabolism and immunity are closely related to PE.

Indeed, we found that the ferroptosis scores of patients with PE were significantly lower than those of women undergoing a normal pregnancy, indicating that iron-mediated cell death is involved in PE development. We subsequently found six ferroptosis-related genes associated with PE by intersecting the genes obtained by WGCNA, DEGs analysis, and reported ferroptosis-related genes. Through the ROC curve, we found that five genes—*BACH1, GCH1, HIF1A, NOS2*, and *PTGS2*—can effectively distinguish between healthy and PE samples, which could be potential PE diagnostic biomarkers.

Hui et al. [29] reported that miR-133a-3p could relieve oxidative stress-induced apoptosis by targeting *BACH1* via regulating the BACH1/NRF2/HO-1 signaling pathway in trophoblast cells. In mammals, GCH1 is the rate-limiting enzyme in biosynthetic processes, which is involved in the synthesis of tetrahydrobiopterin and the pteridine portion of tetrahydrofolate, playing a crucial role in maintaining inflammatory, neurovascular, and cardiovascular homeostasis [30, 31]. However, no studies have identified a role of GCH1 in PE to date.

The expression of HIF1A, which is a negative regulator of RSL3- and erastin-induced ferroptosis in Calu-1 and HT1080 cells [32], was found to be upregulated in the placenta tissues of patients with PE [33]. Takayuki et al. [34] verified that knockdown of *HIF1A* mRNA could alleviate the syndromes of PE, such as hypertension, organ damage, elevated circulating sFlt-1, and proteinuria, in PE mouse models.

Upregulated *NOS2* expression could produce elevated levels of nitric oxide (NO) over extended periods of time. Li et al. [35] found that an increase of the *NOS2* level in lysosomes may cause the continuous accumulation of NO, which will induce autophagy and result in raising the lysosomal membrane permeabilization to its threshold along with lysosomal lipid peroxidation, finally leading to ferroptosis. NO is required for uterine spiral artery remodeling and trophoblast migration in early pregnancy. Patients with PE exhibit lower plasma levels of NO, indicating that the reduced bioavailability of NO is involved in PE development [36, 37].

PTGS2 is an inducible enzyme brought about by hypoxia and oxidative stress [38, 39]. Although PTGS2 does not regulate ferroptosis, it has been reported that increased PTGS2 levels could be a suitable marker for ferroptosis [40]. The increased expression of PTGS2 indicates an inflammatory response in PE [41, 42].

We used the FPI to identify that *NOS2* and *PTGS2* contribute more substantially to the ferroptosis-related etiology of PE among the six candidate biomarkers. The expression pattern of *NOS2* was consistent in the GSE48424 dataset, and the expression level of *NOS2* in patients with PE was significantly higher than that in women undergoing a normal pregnancy. Collectively, these results suggest that *NOS2* is a key ferroptosis-related gene involved in PE. To explain the relevant mechanisms by which *NOS2* participates in PE, we conducted ssGSEA, which showed that sympathetic nervous system development was significantly enriched in the high-*NOS2* expression group. Furthermore, Lina et al. [43] showed that levels of neuron-specific enolase and S100B, which are two cerebral biomarkers indicating neurological injury, remain elevated 1 year postpartum in PE pregnancies versus normal pregnancies. Other studies have reported that ferroptosis plays a significant role in nervous system development and nerve-related diseases [44–47]. Therefore, we speculate that *NOS2* may participate in PE through ferroptosis-mediated neuronal injury. However, this hypothesis needs to be confirmed through further experimental studies.

There are some limitations of this study. The data we used to construct the diagnostic model were downloaded from the GEO database; thus, larger sample sizes should be analyzed. We found a relationship between ferroptosis-related gene signatures and immune-related biological processes. In this study, we also constructed TFs and miRNAs regulatory networks for the target genes and revealed the potential regulatory mechanisms of the ferroptosis related biomarkers in PE. Although we used RT-qPCR analysis to confirm the expression level of hub genes, further in vitro and in vivo experiments are required for validation and functional analyses.

## Conclusions

In this study, WGCNA method was utilized to explore ferroptosis-related genes as biomarkers for the diagnosis of PE. We identified that *NOS2* may serve as a diagnostic biomarker for PE. Our findings revealed that ferroptosis plays a role in PE etiology, thereby enhancing our knowledge of the molecular mechanisms underlying PE.

## Data Availability

The GSE48424 and GSE98224 mRNA profiles were downloaded from the GEO database (https://www.ncbi.nlm.nih.gov/geo/).

## List of abbreviations

AUC: Area under the curve
DEG: Differentially expressed gene
FC: Fold change
FFA: Free fatty acid
FPI: Ferroptosis potential index
GEO: Gene Expression Omnibus
miRNA: MicroRNA
NO: Nitric oxide
PE: Pre-eclampsia
ROC: Receiver operating characteristic
RT-qPCR: Reverse transcription quantitative polymerase chain reaction
ssGSEA: Single-sample gene set enrichment analysis
TF: Transcription factor
WGCNA: Weighted gene co-expression network analysis
SDS-PAGE: sodium dodecyl sulfate-polyacrylamide gel electrophoresis
PVDF: polyvinylidene fluoride
TBST: Tris-buffered saline and Tween 20
HRP: horseradish peroxidase
